# Associated factors, emergency department visits, and hospitalization days of receiving adjunctive Chinese herbal medicine therapy for patients with non-small cell lung cancer: a nationwide cohort study in Taiwan

**DOI:** 10.3389/fphar.2025.1435541

**Published:** 2025-05-20

**Authors:** Meng-Bin Tang, Wei-Yin Kuo, Pei-Tseng Kung, Wen-Chen Tsai

**Affiliations:** ^1^ Department of Family Medicine, China Medical University Hospital, China Medical University, Taichung, Taiwan; ^2^ Department of Health Policy and Management, Chung Shan Medical University, Taichung, Taiwan; ^3^ Department of Medical Management, Chung Shan Medical University Hospital, Taichung, Taiwan; ^4^ Department of Healthcare Administration, Asia University, Taichung, Taiwan; ^5^ Department of Medical Research, China Medical University Hospital, China Medical University, Taichung, Taiwan; ^6^ Department of Health Services Administration, China Medical University, Taichung, Taiwan

**Keywords:** non-small cell lung cancer, adjunctive Chinese medicine therapy, national health insurance research database, medical utilization, Chinese herbal medicine

## Abstract

**Introduction:**

Cancer prevention and treatment, particularly lung cancer, remain major healthcare challenges in Taiwan and globally. This study investigates factors and healthcare utilization patterns associated with adjunctive Chinese herbal medicine (CHM) therapy in non-small cell lung cancer (NSCLC) patients.

**Methods:**

Using Taiwan’s National Health Insurance Research Database and the Taiwan Cancer Registry, we conducted a retrospective cohort study of non-small cell lung cancer patients diagnosed between 2007 and 2013. Descriptive analysis, propensity score matching, and regression models were employed to assess CHM therapy utilization and its impact on emergency department visits and hospitalization days.

**Results:**

Among 43,122 non-small cell lung cancer patients, 5.76% received adjunctive CHM therapy, with the majority at stage IV cancer. Factors such as cancer stage, age, gender, marital status, education level, monthly salary, degree of urbanization, severity of comorbidity, comorbid diseases, hospital ownership, experience of using Chinese medicine, chemotherapy status, and years of diagnosis are significantly associated with whether NSCLC patients receive adjunctive CHM therapy. Patients receiving adjunctive CHM therapy for 181–365 days reduced emergency department visits by 16% (OR = 0.84, 95%CI: 0.74-0.95) and shortened hospitalization days by 17% (Ratio = 0.83, 95%CI: 0.75-0.91).

**Conclusion:**

Prolonged adjunctive CHM therapy, particularly for 181–365 days, is associated with decreased healthcare utilization among non-small cell lung cancer patients. These findings suggest a potential role for extended CHM therapy in managing NSCLC and warrant consideration by clinical teams.

## Introduction

Cancer has been the leading cause of death among the Taiwanese population for 40 years now. In 2022, the cancer mortality rate was 222.7 per 100,000 population. The top three cancer mortality rates were (1) tracheal, bronchus, and lung cancer; (2) liver and intrahepatic bile duct cancer; and (3) colorectal and anal cancer (Ministry of Health and Welfare, 2023). In March 2021, the World Health Organization (WHO) reported that cancer was the leading cause of global deaths in 2020, resulting in nearly ten million deaths. The three most common types of cancer by incidence in the same year were breast cancer (2.26 million), lung cancer (2.21 million), and colorectal cancer (1.93 million). The top three cancers causing death were lung cancer (1.80 million), colorectal cancer (935,000), and liver cancer (830,000) (Cancer Organization, 2021). Looking at both Taiwan and globally, cancer prevention and treatment are major healthcare priorities, with lung cancer being particularly significant. The most common primary malignant tumor in the lungs is epithelial carcinoma, which can be broadly categorized into small cell lung carcinoma (SCLC) and non-small cell lung carcinoma (NSCLC). Non-small cell lung carcinoma accounts for approximately 85% of lung cancers. Histologically, it is divided into adenocarcinoma, squamous cell carcinoma (SCC), and large cell carcinoma (Medscape, 2021). The main treatment modalities include surgical resection, chemotherapy, radiation therapy, targeted therapy, and immunotherapy. Early-stage lung cancer often responds well to treatment, but the mortality rate remains high in advanced stages (Hirsch et al., 2017).

Cancer patients endure significant physical and psychological distress during the treatment process. The side effects of medications and the fear of cancer recurrence often diminish the effectiveness of treatments. As a result, cancer patients often seek alternative therapies in addition to conventional Western medicine. These may include acupuncture and moxibustion, qigong therapy, herbal medicine, dietary therapy, and other approaches that aim to alleviate symptoms and improve overall wellbeing. Complementary and Alternative Medicine (CAM) is embraced by some cancer patients, with research indicating that, on average, patients use one to two types of CAM, which can enhance their quality of life (Kuo et al., 2018). Given that Traditional Chinese Medicine (TCM) therapy is covered by Taiwan’s National Health Insurance, 62.5% of the population seeks Traditional Chinese Herbal Medicines (TCHM) as part of their treatment regimen (Chen et al., 2007). In today’s world, where Western medicine dominates, TCM is recognized as an important form of CAM. Of course, in many countries in Asia, TCM is one of the mainstream medical systems.

In 2009, a study conducted in Hong Kong surveyed 786 cancer patients regarding their use of TCM therapy. The study found that 57.1% of cancer patients utilized a combination of Chinese and Western medicine. Among patients undergoing chemotherapy, a higher proportion sought TCM therapy compared to those undergoing other forms of Western medical treatment (Lam et al., 2009). In 2018, a cross-sectional survey conducted in mainland China involving 590 cancer patients revealed that younger patients (aged less than 60) and those in the early stages of cancer significantly sought TCM therapy (Sun et al., 2018). In 2020, a study conducted in China involving 1950 cancer patients from the southern region examined the use of TCM therapy. The results indicated that cancer patients with higher levels of education (more than 12 years) tended to opt for TCM therapy. Among those who used TCM, 54.61% primarily used Chinese herbal medicine. Additionally, the majority of TCM users (54.51%) underwent treatment for more than 6 months (Xiong et al., 2020).

Previous studies on healthcare utilization among lung cancer patients have often focused on palliative care in the terminal stages, outpatient, inpatient, and intensive care unit (ICU) utilization rates, as well as the proportions of different treatment modalities. Research conducted in Australia on patients with advanced non-small cell lung cancer found that 83% sought palliative care and 82% sought psychosocial support care. However, seeking both types of care did not provide any survival benefit (Duggan et al., 2019). Research comparing healthcare utilization in the last 6 months of life between lung cancer patients and those with chronic obstructive pulmonary disease (COPD) found that COPD patients had higher rates of outpatient visits and ICU admissions, while lung cancer patients had higher utilization of palliative care (Au et al., 2006). American scholars have studied the healthcare utilization of terminally ill lung cancer patients and found that women are more likely than men to use hospitalization, nursing homes, home care, and end-of-life care (Shugarman et al., 2008). Research conducted in mainland China on lung cancer patients has shown an association between comorbidities and healthcare utilization. Comorbidities increase the annual outpatient and inpatient visits among lung cancer patients, increase the probability of choosing chemotherapy and radiotherapy, but decrease the probability of choosing surgery (Ding et al., 2020). Most studies using TCM and CHM in NSCLC were mainly based on survival analysis and treatment effect. The combined-treatment group had better physical function and role function than the Western medicine group at 6 months (p < 0.05) (Tang et al., 2016). The use of CHM as an adjunctive therapy may reduce the mortality hazard ratio of lung cancer patients (Li et al., 2018).

The above literature suggests that past studies on healthcare utilization among lung cancer patients have lacked discussion on the impact of adjunctive TCM therapy and on the research regarding emergency department visits and length of hospitalization. Emergency department utilization and length of hospitalization are important indicators of medical care. Previous studies have pointed out the factors that cancer patients tend to receive adjunctive TCM therapy, including those who have received chemotherapy, those under the age of 60, those with early-stage cancer, and those with higher education levels (Lam et al., 2009; Sun et al., 2018; Xiong et al., 2020). This study would like to explore the factors associated with NSCLC patients receiving adjunctive TCM treatments in Taiwan.

The purpose of this study is to investigate the factors influencing NSCLC patients' acceptance of adjunctive TCM therapy and to explore the difference in emergency medical utilization and length of hospitalization among NSCLC patients based on the duration of adjunctive TCM therapy.

## Materials and methods

### Data sources

This study adopted a retrospective study design. Data were sourced from the National Health Insurance research databases (NHIRD) from the Health and Welfare Data Science Center of the Ministry of Health and Welfare, covering the period from 2000 to 2018, as well as the Cancer Registry issued by the Health Promotion Administration, the Cause of Death Data issued by the Department of Statistics of the Ministry of Health and Welfare, and the Household Registration Database from the Ministry of the Interior. Currently, the national health insurance coverage rate in Taiwan is 99.82%, making it a representative evidence-based medical database.

### Research participants

The inclusion criteria for the study participants were newly diagnosed NSCLC patients, obtained from the Cancer Registry from 2007 to 2013, with observation follow-up until the end of 2018. The lung cancer codes in the Cancer Registry are based on the International Classification of Diseases for Oncology third edition (ICD-O-3 code): C33∼C34. A total of 76,232 individuals were identified. Exclusion criteria were: (1) unclear cancer diagnosis date and time; (2) small cell carcinoma (ICD-O-3 codes: 8041, 8042, 8043, 8044, 8045); (3) *in situ* carcinoma/lung cancer stage 0; (4) unspecified stage; (5) prior history of other cancers before lung cancer diagnosis because this study focused on NSCLC; (6) age less than 20 years old; (7) no active treatment within 1 year, to avoid interference from patients who did not actively seek treatment; (8) unspecified level of the main treatment institution; (9) death within 3 months (90 days) of diagnosis, because it was impossible to track and observe. After exclusions, a total of 43,122 individuals were included in the study.

**FIGURE 1:**
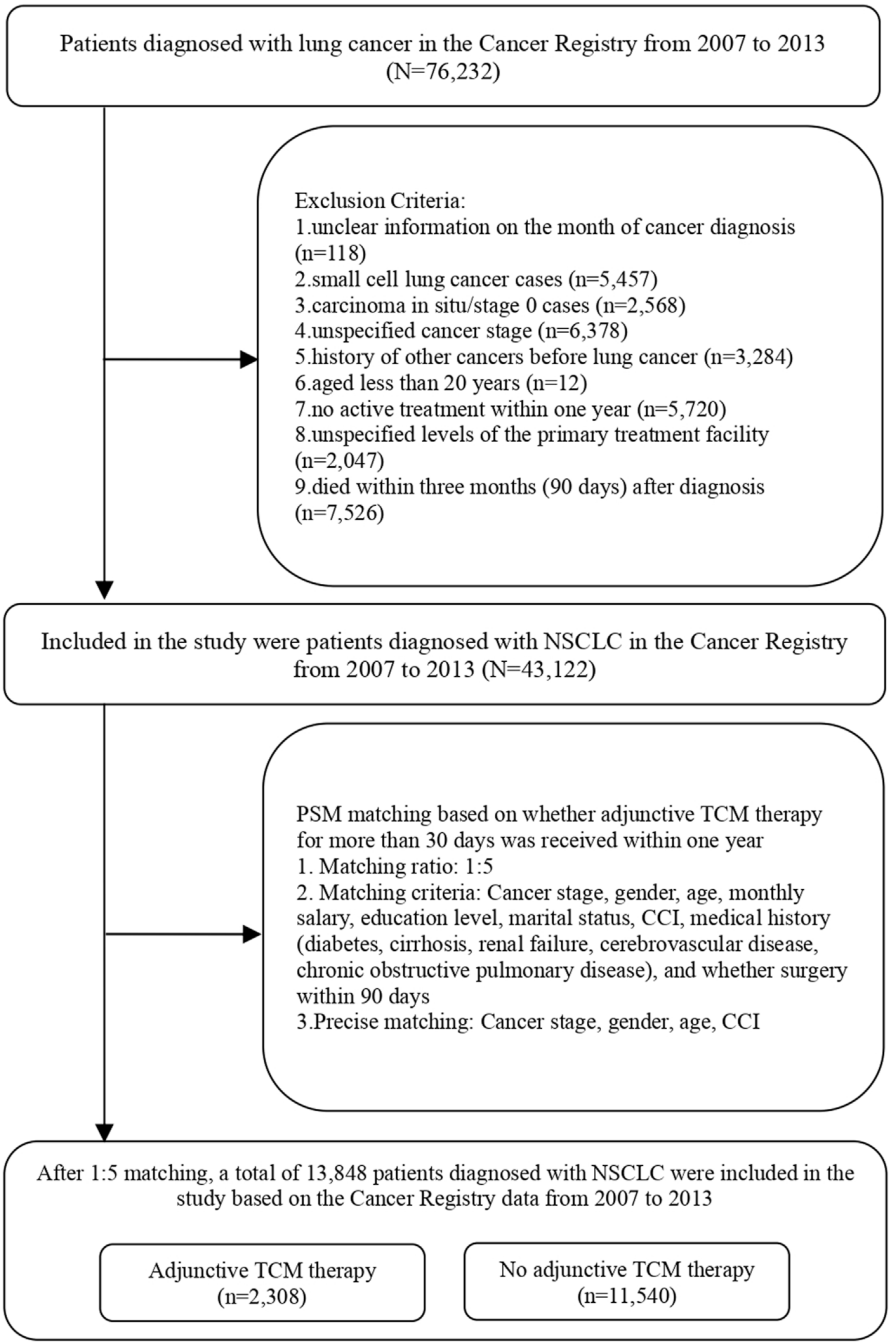
Flow chart.

In order to mitigate the influence of individual characteristics, cancer stage, and disease severity on whether lung cancer patients received adjunctive TCHM therapy, this study employed Propensity Score Matching (PSM) at a ratio of 1:5, taking into account the different personal characteristics, socioeconomic status, comorbidities, and severity of cancer. The matching controlled for variables including cancer stage, gender, age, monthly income, education level, marital status, severity of comorbidity, medical history (diabetes, cirrhosis, renal failure, cerebrovascular disease, chronic obstructive pulmonary disease), cancer diagnosis year, and whether surgery was performed within 90 days. Precise matching was conducted for lung cancer patients who received adjunctive TCHM therapy and those who did not, based on cancer stage, gender, age, and severity of comorbidity. After matching, there were a total of 2,308 lung cancer patients who received adjunctive TCHM therapy and 11,540 lung cancer patients who did not, resulting in a total of 13,848 individuals included in the study analysis (Figure 1; Table 1).

**TABLE 1:** Descriptive analysis before and after matching whether patients with NSCLC receive adjunctive TCM therapy.

Variable		Pre-matching		Adjunctive TCM therapy					Post-matching		Adjunctive TCM therapy				
		Total		No		yes		p value	Total		No		yes		p value
		N	%	N	%	N	%		N	%	N	%	N	%	
Total		43,122	100.00	40,638	94.24	2,484	5.76		13,848	100.00	11,540	83.33	2,308	16.67	
Cancer stage								0.005							1.000
	I	8,002	18.56	7,505	93.79	497	6.21		2,838	20.49	2,365	83.33	473	16.67	
	II	2,240	5.19	2,081	92.90	159	7.10		690	4.98	575	83.33	115	16.67	
	III	9,479	21.98	8,943	94.35	536	5.65		2,940	21.23	2,450	83.33	490	16.67	
	IV	23,401	54.27	22,109	94.48	1,292	5.52		7,380	53.29	6,150	83.33	1,230	16.67	
Age								<0.001							1.000
	20-54 y/o	8,739	20.27	7,939	90.85	800	9.15		4,470	32.28	3,725	83.33	745	16.67	
	55–64 y/o	10,656	24.71	9,859	92.52	797	7.48		4,380	31.63	3,650	83.33	730	16.67	
	65–74 y/o	12,042	27.93	11,450	95.08	592	4.92		3,336	24.09	2,780	83.33	556	16.67	
	≧75 y/o	11,685	27.10	11,390	97.48	295	2.52		1,662	12.00	1,385	83.33	277	16.67	
Gender								<0.001							1.000
	male	24,668	57.21	23,485	95.20	1,183	4.80		6,666	48.14	5,555	83.33	1,111	16.67	
	female	18,454	42.79	17,153	92.95	1,301	7.05		7,182	51.86	5,985	83.33	1,197	16.67	
CCI								0.321							1.000
	0	41,147	95.42	38,792	94.28	2,355	5.72		13,428	96.97	11,190	83.33	2,238	16.67	
	1	1,139	2.64	1,065	93.50	74	6.50		240	1.73	200	83.33	40	16.67	
	≥2	836	1.94	781	93.42	55	6.58		180	1.30	150	83.33	30	16.67	
Surgery within 90 days								0.036							0.223
	no	6,219	14.42	5,897	94.82	322	5.18		1,733	12.51	1,426	82.29	307	17.71	
	yes	36,903	85.58	34,741	94.14	2,162	5.86		12,115	87.49	10,114	83.48	2,001	16.52	
Marital status								<0.001							0.588
	Married	30,033	69.65	28,097	93.55	1,936	6.45		11,054	79.82	9,233	83.53	1,821	16.47	
	Unmarried	1,919	4.45	1,810	94.32	109	5.68		613	4.43	507	82.71	106	17.29	
	Divorced/separated	2,732	6.34	2,592	94.88	140	5.12		730	5.27	597	81.78	133	18.22	
	Widowed	6,617	15.34	6,361	96.13	256	3.87		1,451	10.48	1,203	82.91	248	17.09	
	missing	1,821	4.22		0.00		0.00								
Education level								<0.001							0.545
	Elementary school or below	21,037	48.78	20,216	96.10	821	3.90		4,838	34.94	4,038	83.46	800	16.54	
	Junior high school	6,280	14.56	5,901	93.96	379	6.04		2,302	16.62	1,938	84.19	364	15.81	
	High school (vocational)	7,704	17.87	7,075	91.84	629	8.16		3,487	25.18	2,898	83.11	589	16.89	
	College and above	6,285	14.57	5,673	90.26	612	9.74		3,221	23.26	2,666	82.77	555	17.23	
	missing	1,923	4.46		0.00		0.00								
Monthly salary (NT$)								<0.001							0.959
	≤17,280	11,144	25.84	10,630	95.39	514	4.61		2,944	21.26	2,461	83.59	483	16.41	
	17,281–22,800	17,231	39.96	16,381	95.07	850	4.93		4,840	34.95	4,042	83.51	798	16.49	
	22,801–36,300	6,018	13.96	5,593	92.94	425	7.06		2,418	17.46	2,011	83.17	407	16.83	
	36,301–57,800	5,375	12.46	4,953	92.15	422	7.85		2,275	16.43	1,890	83.08	385	16.92	
	≥57,801	3,140	7.28	2,888	91.97	252	8.03		1,371	9.90	1,136	82.86	235	17.14	
	missing	214	0.50		0.00		0.00								
Comorbidities															
	Diabetes	3,548	8.23	3,443	97.04	105	2.96	<0.001	523	3.78	424	81.07	99	18.93	0.175
	COPD	2,252	5.22	2,195	97.47	57	2.53	<0.001	276	1.99	223	80.80	53	19.20	0.289
	CVD	1,287	2.98	1,239	96.27	48	3.73	0.002	227	1.64	182	80.18	45	19.82	0.231
	Liver cirrhosis	563	1.31	540	95.91	23	4.09	0.104	97	0.70	74	76.29	23	23.71	0.083
	Renal failure	387	0.90	382	98.71	5	1.29	<0.001	14	0.10	12	85.71	2	14.29	0.550

*control variables including cancer stage, gender, age, salary, education level, marital status, severity of comorbidities, diabetes, liver cirrhosis, renal failure, cerebrovascular disease, chronic obstructive pulmonary disease and whether surgery was performed within 90 days; precise matching of lung cancer patients who received TCM, as adjunctive therapy with those who did not in terms of cancer stage, gender, age, and severity of comorbidities.

**CCI: charlson comorbidity index; NT$: new taiwan dollar.

### Variable definition and explanation

The inclusion criteria for receiving adjunctive TCHM therapy were defined as follows: The first diagnosis code for lung cancer during the visit to a Chinese medicine practitioner must be lung cancer. Patients who used adjunctive TCHM therapy for less than 30 days within 1 year after diagnosis were defined as non-users of adjunctive TCHM therapy, while those who used adjunctive TCHM therapy for 30 days or more within 1 year after diagnosis were defined as users (Lin et al., 2015). Subsequently, the use of adjunctive TCHM therapy within 1 year after diagnosis was further divided into three groups: <30 days, 30–180 days, and 181–365 days. The experience of using Chinese medicine referred to whether patients had visited a Chinese medicine department in the 2 years before being diagnosed with NSCLC. Emergency department utilization by patients referred to whether NSCLC patients visited the emergency department within 1 year after diagnosis. The total number of hospitalization days for patients referred to the total number of hospitalization days within 1 year after diagnosis of NSCLC. When we analyzed the emergency department utilization of patients with NSCLC, regardless of whether the diagnosis of the emergency department visits was lung cancer, if there were emergency department visits, they had been counted. Because NSCLC and its treatment have many complications, any emergency visits may be related to NSCLC or treatments.

Basic personal information includes gender, age, marital status, and education level. Gender is divided into male and female. Age is divided into five age groups: 20–54 years old, 55–64 years old, 65–74 years old, ≥75 years old (Huang et al., 2014). Marital status is categorized as married, divorced/separated, widowed, or unmarried. Education level is classified as elementary school or below, junior high school, high school (including vocational), and college or above. Economic status is grouped based on monthly salary, ranging from high to low: ≥57,801 NT dollars, 36,301–57800 NT dollars, 22,801–36300 NT dollars, 17,281–22800 NT dollars, and ≤17,280 NT dollars. Environmental factors are differentiated by the degree of urbanization in the residential area, categorized according to the urbanization levels proposed by Liu (2006) et al., which divided Taiwan into seven levels of urbanization, from the highest urbanization level (Level 1) to the lowest (Level 7) (Liu et al., 2006). Due to the small number of individuals in Level 6 and Level 7, this study combined them with Level 5.

The comorbidities were based on whether the primary or secondary diagnosis codes in the medical records of patients in the 2 years before the diagnosis of NSCLC include the following disease codes, with the disease diagnosis occurring two or more times. Hypertension (ICD-9 codes 401-405; ICD-10 codes I10-I15), diabetes (ICD-9 code 250; ICD-10 codes E08-E13), hyperlipidemia (ICD-9 code 272; ICD-10 codes E78), liver cirrhosis (ICD-9 code 571; ICD-10 codes K70, K73-K76), renal failure (ICD-9 codes 584-586; ICD-10 codes N17-N19), cerebral vascular disease (ICD-9 codes 430-438; ICD-10 codes I60-I69), chronic obstructive pulmonary disease (ICD-9 codes 491, 492, and 496; ICD-10 codes J41-J44), asthma (ICD-9 codes 493; ICD-10 codes J45), rheumatoid arthritis (ICD-9 codes 714; ICD-10 codes M05-M06), and chronic mental disorder (ICD-9 codes 295, 296; ICD-10 codes F20, F30-F34, F39). The severity of comorbidities adopts the Charlson Comorbidity Index (CCI) as defined by Deyo et al. (1992). The original disease categories of the CCI are defined based on the diagnosis or procedure codes of ICD-9-CM, where a higher score indicates a more severe comorbidity level. The CCI score is calculated by converting the primary or secondary diagnosis codes from medical visits in the 2 years before diagnosis into scores, and the cumulative score represents the comorbidity level.

Hospital attributes refer to the institution where the first treatment (surgery, chemotherapy, radiotherapy, targeted therapy) for lung cancer diagnosis within 6 months is received. For those without treatment, it refers to the institution where the primary diagnosis first occurred. Hospital ownership is classified as public and non-public institutions. Hospital levels include medical centers, regional hospitals, and district hospitals. Cancer staging follows the TNM (tumor-node-metastasis) system and is categorized into stages I, II, III, and IV (Kay et al., 2017). Staging data is primarily obtained from the Cancer Registry. Treatment modalities include surgery, chemotherapy, radiotherapy, targeted therapy, surgery combined with other treatments, chemotherapy combined with other treatments, and other treatment combinations. NSCLC diagnosis years range from 2007 to 2013.

### Statistical analysis

This study utilized statistical software SAS 9.4 (SAS Institute Inc., Cary, NC, USA) to analyze and process the data, conducting both descriptive and inferential statistics. All statistical analyses were considered significant at the p < 0.05 level and described as follows. Descriptive statistics were used to describe frequencies, percentages, means, etc. Specifically, descriptive analysis was conducted before and after matching on whether NSCLC patients received adjunctive TCHM therapy. It included the proportions of NSCLC patients with and without adjunctive TCHM therapy across different demographic variables, socioeconomic status, environmental factors, health conditions, cancer stages, hospital attributes, treatment modalities, year of diagnosis, etc.

Inferential statistics include exploring the factors influencing whether NSCLC patients receive adjunctive TCHM therapy and investigating the differences in emergency department visits and hospitalization days among NSCLC patients receiving adjunctive TCHM therapy. The Chi-Square test was employed to compare the differences in receiving or not receiving adjunctive TCHM therapy with gender, age, marital status, education level, monthly salary, degree of urbanization of residential area, severity of comorbidities, comorbid diseases, cancer stage, hospital level, ownership, treatment modalities, and year of diagnosis. Logistic regression analysis was conducted to investigate the factors influencing whether NSCLC patients receive adjunctive TCHM therapy. The dependent variable was the receipt of adjunctive TCHM therapy, and the independent variables included gender, age, marital status, education level, monthly salary, degree of urbanization of residential area, comorbid diseases, cancer stage, hospital level, ownership, treatment modalities, and year of diagnosis.

For the analysis of the association between adjunctive TCHM therapy and emergency department visits, conditional logistic regression can be employed. To analyze the association between hospitalization days of NSCLC patients and receiving adjunctive TCHM therapy, multiple regression analysis with Generalized Estimating Equations (GEE) was utilized. Since the distribution of hospitalization days for NSCLC patients within 1 year is not normally distributed, the hospitalization days of the study subjects will be transformed using a natural logarithm to approximate a normal distribution before conducting the GEE multiple regression analysis.

This study was reviewed and approved by the Research Ethics Center of China Medical University Hospital (IRB No. CMUH110-REC2-244). This research used unidentifiable data obtained from government legal databases (e.g., NHIRD, Cancer Registry). Therefore, patient consent is not required to access the NHIRD. The authors assert that all procedures contributing to this work comply with the ethical standards of the relevant national and institutional committees on human experimentation and with the Helsinki Declaration of 1975, as revised in 2008.

## Results

This study included 43,122 patients with NSCLC, of whom 2,484 received adjunctive TCHM therapy, accounting for only 5.76% of the total. Among the cancer stages, the highest number of patients was in stage IV, with 23,401 individuals (54.27%). In terms of age distribution, the majority were aged 65–74 years (27.93%) and those aged 75 years and above (27.10%). Among the patients, there were 24,668 males (57.21%) and 18,454 females (42.79%). Regarding marital status, the majority were married, accounting for 69.65%, while in terms of education level, the highest proportion had education up to or below elementary school (48.78%). Among the patients, 41,147 individuals (95.42%) had a CCI score of 0 for comorbidities, and among those with comorbidities, hypertension was the most common, accounting for 15.82%. In terms of treatment methods, the highest number of patients had undergone surgical treatment, accounting for 93.16%.

The factors significantly associated with whether NSCLC patients receive adjunctive TCHM therapy (as shown in Table 2) include cancer stage, age, gender, marital status, education level, monthly salary, degree of urbanization of residential area, severity of comorbidities, comorbid diseases, hospital ownership, experience with Chinese medicine, receipt of chemotherapy, and year of diagnosis (p < 0.05). Compared to patients in stage I, those in stages II and III have a higher probability of receiving adjunctive TCHM therapy (OR = 1.35, 95% CI: 1.11-1.65; OR = 1.23, 95% CI: 1.06-1.41). Elderly patients aged 65–74 years and those aged 75 years and above are less likely to receive adjunctive TCHM therapy compared to the 20-54 age group (OR = 0.80, 95% CI: 0.70-0.92; OR = 0.46, 95% CI: 0.39-0.55). Females are significantly more likely than males to seek adjunctive TCHM therapy (OR = 1.37, 95% CI: 1.25-1.50). Married patients are more likely to receive adjunctive TCHM therapy compared to unmarried, divorced/separated, or widowed patients. Regarding education level, patients with higher levels of education are more inclined to use adjunctive TCHM therapy, with those having a college degree or above being more likely to use it compared to those with elementary school education or below (OR = 2.17, 95% CI: 1.89-2.48). Patients with higher monthly salaries (≥57,801 NT$) are more likely to use adjunctive TCHM therapy compared to those with the lowest monthly salary (≤17,280 NT$) (OR = 1.22, 95% CI: 1.04-1.44). Patients with a CCI score ≥2 are more likely to receive adjunctive TCHM therapy compared to those with a CCI score of 0 (OR = 1.36, 95% CI: 1.02-1.82). However, patients with certain comorbidities are less likely to use adjunctive TCHM therapy, such as those with hypertension (OR = 0.83, 95% CI: 0.72-0.96), diabetes (OR = 0.67, 95% CI: 0.54-0.83), renal failure (OR = 0.36, 95% CI: 0.15-0.88), or chronic obstructive pulmonary disease (OR = 0.72, 95% CI: 0.55-0.96) compared to those without these comorbidities. Patients in public hospitals are less likely to receive adjunctive TCHM therapy compared to those in non-public hospitals (OR = 0.90, 95% CI: 0.82-0.99). Patients with prior experience with Chinese medicine are significantly more likely to receive adjunctive TCHM therapy compared to those without such experience (OR = 1.94, 95% CI: 1.78-2.11). Patients who have undergone chemotherapy are significantly more likely to seek adjunctive TCHM therapy compared to those who have not (OR = 1.13, 95% CI: 1.03-1.25). Patients diagnosed in later years are more likely to seek adjunctive TCHM therapy (ORs: 1.28–2.18). This analysis reveals that only hospital level does not significantly affect patients' receipt of adjunctive TCHM therapy (p > 0.05).

**TABLE 2:** Related factors affecting NSCLC patients receiving adjunctive TCM therapy.

Variable		Adjunctive TCM therapy										
		Total		No		yes		p value	OR	95% CI		p value
		N	%	N	%	N	%					
Total		43,122	100.00	40,638	94.24	2,484	5.76					
Cancer stage								0.005				
	I	8,002	18.56	7,505	93.79	497	6.21		1.00			
	II	2,240	5.19	2,081	92.90	159	7.10		1.35	1.11	1.65	0.003
	III	9,479	21.98	8,943	94.35	536	5.65		1.23	1.06	1.41	0.005
	IV	23,401	54.27	22,109	94.48	1,292	5.52		1.05	0.93	1.18	0.471
Age								<0.001				
	20-54 y/o	8,739	20.27	7,939	90.85	800	9.15		1.00			
	55–64 y/o	10,656	24.71	9,859	92.52	797	7.48		0.97	0.87	1.09	0.603
	65–74 y/o	12,042	27.93	11,450	95.08	592	4.92		0.80	0.70	0.92	0.001
	≧75 y/o	11,685	27.10	11,390	97.48	295	2.52		0.46	0.39	0.55	<0.001
Gender								<0.001				
	Male	24,668	57.21	23,485	95.20	1,183	4.80		1.00			
	Female	18,454	42.79	17,153	92.95	1,301	7.05		1.37	1.25	1.50	<0.001
Marital status								<0.001				
	Married	30,033	69.65	28,097	93.55	1,936	6.45		1.00			
	Unmarried	1,919	4.45	1,810	94.32	109	5.68		0.69	0.56	0.85	0.001
	Divorced/separated	2,732	6.34	2,592	94.88	140	5.12		0.64	0.54	0.77	<0.001
	Widowed	6,617	15.34	6,361	96.13	256	3.87		0.82	0.71	0.95	0.009
	Miss	1,821	4.22									
Education level								<0.001				
	Elementary school or below	21,037	48.78	20,216	96.10	821	3.90		1.00			
	Junior high school	6,280	14.56	5,901	93.96	379	6.04		1.29	1.13	1.48	<0.001
	High school (vocational)	7,704	17.87	7,075	91.84	629	8.16		1.73	1.53	1.95	<0.001
	College and above	6,285	14.57	5,673	90.26	612	9.74		2.17	1.89	2.48	<0.001
	Miss	1,923	4.46									
Monthly salary (NT$)								<0.001				
	≤17,280	11,144	25.84	10,630	95.39	514	4.61		1.00			
	17,281–22,800	17,231	39.96	16,381	95.07	850	4.93		1.08	0.95	1.22	0.230
	22,801–36,300	6,018	13.96	5,593	92.94	425	7.06		1.17	1.02	1.35	0.029
	36,301–57,800	5,375	12.46	4,953	92.15	422	7.85		1.25	1.09	1.44	0.002
	≥57,801	3,140	7.28	2,888	91.97	252	8.03		1.22	1.04	1.44	0.018
	Miss	214	0.50									
Degree of urbanization								<0.001				
	1	11,565	26.82	10,878	94.06	687	5.94		1.00			
	2	12,636	29.30	11,807	93.44	829	6.56		1.18	1.06	1.31	0.003
	3	6,761	15.68	6,367	94.17	394	5.83		1.12	0.98	1.27	0.110
	4	6,666	15.46	6,336	95.05	330	4.95		1.05	0.91	1.22	0.484
	5	5,280	12.24	5,057	95.78	223	4.22		1.08	0.91	1.28	0.382
	Miss	214	0.50									
CCI								0.321				
	0	41,147	95.42	38,792	94.28	2,355	5.72		1.00			
	1	1,139	2.64	1,065	93.50	74	6.50		1.09	0.85	1.40	0.489
	≥2	836	1.94	781	93.42	55	6.58		1.36	1.02	1.82	0.035
Comorbidities												
Hypertension								<0.001				
	No	36,302	84.18	34,073	93.86	2,229	6.14		1.00			
	Yes	6,820	15.82	6,565	96.26	255	3.74		0.83	0.72	0.96	0.012
Diabetes								<0.001				
	No	39,574	91.77	37,195	93.99	2,379	6.01		1.00			
	Yes	3,548	8.23	3,443	97.04	105	2.96		0.67	0.54	0.83	<0.001
Hyperlipidemia								0.140				
	No	42,129	97.70	39,691	94.21	2,438	5.79		1.00			
	Yes	993	2.30	947	95.37	46	4.63		1.03	0.75	1.41	0.853
Liver cirrhosis								0.104				
	No	42,559	98.69	40,098	94.22	2,461	5.78		1.00			
Renal failure								<0.001				
	No	42,735	99.10	40,256	94.20	2,479	5.80		1.00			
	Yes	387	0.90	382	98.71	5	1.29		0.36	0.15	0.88	0.025
CVD								0.002				
	No	41,835	97.02	39,399	94.18	2,436	5.82		1.00			
	Yes	1,287	2.98	1,239	96.27	48	3.73		1.05	0.77	1.43	0.753
COPD								<0.001				
	No	40,870	94.78	38,443	94.06	2,427	5.94		1.00			
	Yes	2,252	5.22	2,195	97.47	57	2.53		0.72	0.55	0.96	0.023
Asthma								0.265				
	No	42,460	98.46	40,007	94.22	2,453	5.78		1.00			
	Yes	662	1.54	631	95.32	31	4.68		1.04	0.72	1.51	0.837
Rheumatoid arthritis								1.000				
	No	43,023	99.77	40,545	94.24	2,478	5.76		1.00			
	Yes	99	0.23	93	93.94	6	6.06		1.13	0.49	2.62	0.782
chronic mental disorder								0.236				
	No	42,967	99.64	40,488	94.23	2,479	5.77		1.00			
	Yes	155	0.36	150	96.77	5	3.23		0.58	0.23	1.42	0.231
Hospital ownership								0.792				
	Non-public	26,844	62.25	25,291	94.21	1,553	5.79		1.00			
	Public	16,278	37.75	15,347	94.28	931	5.72		0.90	0.82	0.99	0.022
Hospital level								0.042				
	Medical rights	27,730	64.31	26,077	94.04	1,653	5.96		1.00			
	Regional hospitals	14,443	33.49	13,658	94.56	785	5.44		1.01	0.92	1.11	0.861
	District hospitals	949	2.20	903	95.15	46	4.85		1.07	0.79	1.46	0.663
TCM experience								<0.001				
	No	24,126	55.95	23,189	96.12	937	3.88		1.00			
	Yes	18,996	44.05	17,449	91.86	1,547	8.14		1.94	1.78	2.11	<0.001
Surgery								0.979				
	No	2,948	6.84	2,779	94.27	169	5.73		1.00			
	Yes	40,174	93.16	37,859	94.24	2,315	5.76		0.92	0.77	1.09	0.344
Chemotherapy								0.067				
	No	18,075	41.92	17,078	94.48	997	5.52		1.00			
	Yes	25,047	58.08	23,560	94.06	1,487	5.94		1.13	1.03	1.25	0.015
Radiotherapy								0.105				
	No	32,282	74.86	30,457	94.35	1,825	5.65		1.00			
	Yes	10,840	25.14	10,181	93.92	659	6.08		1.01	0.91	1.12	0.850
Targeted therapy								0.025				
	No	19,817	45.96	18,621	93.96	1,196	6.04		1.00			
	Yes	23,305	54.04	22,017	94.47	1,288	5.53		1.04	0.93	1.16	0.493
Other treatment								0.532				
	No	43,078	99.90	40,598	94.24	2,480	5.76		1.00			
	Yes	44	0.10	40	90.91	4	9.09		1.42	0.49	4.10	0.514
Years of diagnosis								<0.001				
	2007	5,290	12.27	5,098	96.37	192	3.63		1.00			
	2008	5,281	12.25	5,032	95.28	249	4.72		1.28	1.05	1.55	0.015
	2009	5,989	13.89	5,663	94.56	326	5.44		1.50	1.25	1.81	<0.001
	2010	6,168	14.30	5,839	94.67	329	5.33		1.49	1.23	1.79	<0.001
	2011	6,456	14.97	6,069	94.01	387	5.99		1.85	1.53	2.24	<0.001
	2012	6,984	16.20	6,489	92.91	495	7.09		2.18	1.82	2.62	<0.001
	2013	6,954	16.13	6,448	92.72	506	7.28		2.03	1.69	2.43	<0.001

*OR: odds ratio; CCI: charlson comorbidity index; NT$: new taiwan dollar; y/o: years old; CI: confidence interval; CVD: cerebral vascular disease.

In this study, adjunctive TCM therapy primarily focuses on exploring herbal medicine. Table 3 presents the 20 most commonly used single herb and formula among NSCLC patients. There are 439 single herb medicine items, totaling 440,971 prescription records, and 456 formula items, totaling 251,750 prescription records. The top 10 most commonly used single herb medicines are Hedyotis diffusa, Platycodon root, Astragalus root, Tendrilleaf fritillary bulb, Heartleaf houttuynia, Skullcap, Scutellaria root, Bitter apricot seed, Rhubarb, and Mongolian dandelion. The top 10 most commonly used formulas are San Jhong Kuei Jian Tang, Siang Sha Liou Jyun Zih Tang, Xiao Chai Hu Tang, Jiawei Siaoyao San, Bai He Gu Jin Tang, Qing Zao Jiu Fei Tang, Bansia Siexin Tang, Bu Zhong Yi Qi Tang, Ganlu Yin, and Wen Dan Tang. Hedyotis diffusa accounts for 4.23% of all prescriptions, making it significantly higher than other single herb medicines, while San Jhong Kuei Jian Tang accounts for 1.73% of all prescriptions, making it the most prescribed formula, slightly higher than the second most prescribed Siang Sha Liou Jyun Zih Tang (1.41%).

**TABLE 3:** The most common TCM herbs and formula used by NSCLC patients.

Order	Herb	N	%	Order	Formula	N	%
1	Hedyotis diffusa	18,652	4.23	1	San Jhong Kuei Jian Tang	7,622	1.73
2	Platycodon root	8,965	2.03	2	Siang Sha Liou Jyun Zih Tang	6,239	1.41
3	Astragalus root	8,340	1.89	3	Xiao Chai Hu Tang	5,758	1.31
4	Tendrilleaf fritillary bulb	8,333	1.89	4	Jiawei Siaoyao San	5,742	1.30
5	Heartleaf houttuynia	7,950	1.80	5	Bai He Gu Jin Tang	5,494	1.25
6	Skullcap	7,907	1.79	6	Qing Zao Jiu Fei Tang	5,329	1.21
7	Scutellaria root	6,898	1.56	7	Bansia Siexin Tang	5,306	1.20
8	Bitter apricot seed	6,692	1.52	8	Bu Zhong Yi Qi Tang	4,488	1.02
9	Rhubarb	6,183	1.40	9	Ganlu Yin	4,278	0.97
10	Mongolian dandelion	6,148	1.39	10	Wen Dan Tang	4,233	0.96
11	Magnolia bark	5,826	1.32	11	Mai Men Dong Tang	3,959	0.90
12	Red sage root and rhizome	5,813	1.32	12	Xue Fu Zhu Yu Tang	3,872	0.88
13	White atractylodes rhizome	5,694	1.29	13	Ping Wei San	3,748	0.85
14	Indian bread	5,533	1.25	14	Zhi Gan Cao Tang	3,748	0.85
15	Pinellia tuber	5,141	1.17	15	Sha Shen Mai Dong Tang	3,621	0.82
16	Dwarf lilyturf root	5,001	1.13	16	Ma Xing Gan Shi Tang	3,276	0.74
17	Corydalis tuber	4,955	1.12	17	Sheng Mai Yin	3,220	0.73
18	Pepperweed seed Tansymustard seed	4,604	1.04	18	Ding Chuan Tang	3,209	0.73
19	Liquorice root and rhizome	4,211	0.95	19	Xin Yi Qing Fei Tang	3,086	0.70
20	Pilose asiabell root	3,986	0.90	20	Xiao Qing Long Tang	2,980	0.68

In total, 43,122 patients with NSCLC were enrolled in the study. Among them, 40,638 patients (94.24%) did not receive adjunctive TCHM therapy, while 2,484 patients did. To mitigate data bias and confounding variables, we employed Propensity Score Matching (PSM) at a 1:5 ratio to precisely match cancer stage, age, gender, and severity of comorbidities. After matching, there were 2,308 patients who received adjunctive TCHM therapy and 11,540 patients who did not. The matched variables included cancer stage, age, gender, monthly salary, education level, marital status, severity of comorbidities, diabetes, cirrhosis, renal failure, cerebrovascular disease, chronic obstructive pulmonary disease, and whether surgery was received within 90 days of diagnosis. The differences between patients receiving and not receiving adjunctive TCHM therapy were not significant (p > 0.05) after matching (see Figure 1; Table 1).

Table 4 presents the emergency department visits within 1 year of diagnosis for patients with NSCLC and its relevant factors. 57.06% of NSCLC patients had emergency department visits within 1 year of diagnosis. Patients who received adjunctive TCM therapy for 181–365 days had significantly lower emergency department visits (45.45%) compared to those who did not receive adjunctive TCHM therapy (57.31%) (OR = 0.84, 95% CI: 0.74-0.95).

**TABLE 4:** Emergency department visit and related factors among NSCLC patients within 1 year of diagnosis.

Variable				Emergency department visit				p value	Adjusted model			
		Total		No		yes						
		N	%	N	%	N	%		OR	95%CI		p value
Total		13,848	100.00	5,947	42.94	7,901	57.06					
Days of TCM								<0.001				
	<30	11,540	83.33	4,927	42.69	6,613	57.31					
	30-180	1,692	12.22	684	40.43	1,008	59.57		1.02	0.96	1.09	0.539
	181-365	616	4.45	336	54.55	280	45.45		0.84	0.74	0.95	0.004
Cancer stage								<0.001				
	I	2,838	20.49	1,945	68.53	893	31.47					
	II	690	4.98	330	47.83	360	52.17		1.54	1.36	1.75	<0.001
	III	2,940	21.23	1,120	38.10	1,820	61.90		1.86	1.71	2.02	<0.001
	IV	7,380	53.29	2,552	34.58	4,828	65.42		2.03	1.89	2.19	<0.001
Age								<0.001				
	20-54 y/o	4,470	32.28	2,084	46.62	2,386	53.38					
	55–64 y/o	4,380	31.63	1,948	44.47	2,432	55.53		0.99	0.93	1.05	0.776
	65–74 y/o	3,336	24.09	1,364	40.89	1,972	59.11		1.01	0.94	1.08	0.880
	≧75 y/o	1,662	12.00	551	33.15	1,111	66.85		1.11	1.02	1.21	0.020
Gender								<0.001				
	male	6,666	48.14	2,604	39.06	4,062	60.94					
	female	7,182	51.86	3,343	46.55	3,839	53.45		0.89	0.85	0.94	<0.001
Marital status								<0.001				
	Unmarried	613	4.43	290	47.31	323	52.69		1.00	0.89	1.12	0.994
	Married	11,054	79.82	4,806	43.48	6,248	56.52					
	Divorced/separated	730	5.27	290	39.73	440	60.27		1.12	1.01	1.23	0.030
	Widowed	1,451	10.48	561	38.66	890	61.34		1.01	0.94	1.09	0.820
Education level								<0.001				
	Elementary school or below	4,838	34.94	1,811	37.43	3,027	62.57					
	Junior high school	2,302	16.62	885	38.44	1,417	61.56		0.99	0.92	1.05	0.659
	High school (vocational)	3,487	25.18	1,570	45.02	1,917	54.98		0.91	0.85	0.97	0.005
	College and above	3,221	23.26	1,681	52.19	1,540	47.81		0.84	0.78	0.90	<0.001
Monthly salary (NT$)								<0.001				
	≤17,280	2,944	21.26	1,203	40.86	1,741	59.14					
	17,281–22,800	4,840	34.95	1,908	39.42	2,932	60.58		0.97	0.91	1.03	0.313
	22,801–36,300	2,418	17.46	1,041	43.05	1,377	56.95		0.97	0.90	1.04	0.412
	36,301–57,800	2,275	16.43	1,026	45.10	1,249	54.90		0.96	0.89	1.03	0.259
	≥57,801	1,371	9.90	769	56.09	602	43.91		0.84	0.77	0.93	0.001
Degree of urbanization								<0.001				
	1	4,166	30.08	1,920	46.09	2,246	53.91					
	2	4,357	31.46	1,900	43.61	2,457	56.39		1.01	0.95	1.07	0.756
	3	2,108	15.22	872	41.37	1,236	58.63		1.02	0.95	1.10	0.561
	4	1,899	13.71	747	39.34	1,152	60.66		1.05	0.97	1.13	0.251
	5	1,318	9.52	508	38.54	810	61.46		1.01	0.93	1.10	0.805
CCI								0.047				
	0	13,428	96.97	5,782	43.06	7,646	56.94					
	1	240	1.73	104	43.33	136	56.67		1.03	0.87	1.22	0.771
	≥2	180	1.30	61	33.89	119	66.11		1.05	0.87	1.26	0.633
Comorbidities												
	hypertension	1,533	11.07	552	36.01	981	63.99	<0.001	1.06	0.99	1.14	0.094
	diabetes	523	3.78	168	32.12	355	67.88	<0.001	1.06	0.94	1.18	0.348
	hyperlipidemia	238	1.72	99	41.60	139	58.40	0.721	0.98	0.82	1.16	0.784
	liver cirrhosis	97	0.70	26	26.80	71	73.20	0.002	1.21	0.96	1.53	0.116
	renal failure	14	0.10	2	14.29	12	85.71	0.058	1.25	0.71	2.22	0.438
	CVD	227	1.64	57	25.11	170	74.89	<0.001	1.15	0.98	1.34	0.089
	COPD	276	1.99	64	23.19	212	76.81	<0.001	1.17	1.02	1.35	0.027
	asthma	178	1.29	58	32.58	120	67.42	0.006	1.11	0.92	1.33	0.270
	rheumatoid arthritis	33	0.24	12	36.36	21	63.64	0.556	1.11	0.72	1.71	0.631
	chronic mental disorder	38	0.27	11	28.95	27	71.05	0.114	1.19	0.81	1.73	0.382
Hospital ownership								<0.001				
	non-public	8,381	60.52	3,479	41.51	4,902	58.49					
	public	5,467	39.48	2,468	45.14	2,999	54.86		1.04	0.99	1.09	0.108
Hospital level								<0.001				
	medical centers	9,365	67.63	4,149	44.30	5,216	55.70					
	regional hospitals	4,246	30.66	1,716	40.41	2,530	59.59		1.00	0.95	1.05	0.929
	district hospitals	237	1.71	82	34.60	155	65.40		1.00	0.85	1.17	0.991
TCM experience								0.270				
	No	6,968	50.32	3,025	43.41	3,943	56.59					
	yes	6,880	49.68	2,922	42.47	3,958	57.53		1.01	0.97	1.06	0.606
Treatment modalities								<0.001				
	Surgery	3,383	24.43	1,611	47.62	1,772	52.38		1.91	1.43	2.57	<0.001
	Chemotherapy	79	0.57	45	56.96	34	43.04		1.29	0.82	2.00	0.269
	Radiotherapy	98	0.71	47	47.96	51	52.04		1.61	1.08	2.41	0.019
	Targeted therapy	133	0.96	87	65.41	46	34.59		1.00			
	Surgery + others	9,601	69.33	3,880	40.41	5,721	59.59		1.93	1.44	2.58	<0.001
	Chemotherapy + others	418	3.02	206	49.28	212	50.72		1.48	1.07	2.04	0.017
	Other combinations	136	0.98	71	52.21	65	47.79		1.42	0.97	2.08	0.068
Years of diagnosis								<0.001				
	2007	1,616	11.67	622	38.49	994	61.51					
	2008	1,694	12.23	678	40.02	1,016	59.98		1.00	0.92	1.09	0.990
	2009	1,925	13.90	816	42.39	1,109	57.61		0.98	0.90	1.06	0.565
	2010	1,989	14.36	805	40.47	1,184	59.53		1.00	0.92	1.09	0.949
	2011	1,967	14.20	855	43.47	1,112	56.53		0.98	0.90	1.07	0.679
	2012	2,182	15.76	1,036	47.48	1,146	52.52		0.94	0.86	1.02	0.156
	2013	2,475	17.87	1,135	45.86	1,340	54.14		0.97	0.89	1.05	0.415

In terms of other relevant factors, the cancer stage of NSCLC patients is directly proportional to emergency department visits (ORs: 1.54–2.03). Patients aged 75 years and above have more emergency department visits compared to those aged 20–54 years (OR = 1.11, 95% CI: 1.02-1.21). Female patients have fewer emergency department visits compared to male patients (OR = 0.89, 95% CI: 0.85-0.94). Regarding education level, patients with a high school (vocational) education and those with a college degree or above significantly utilize emergency department services fewer than those with elementary school education or below (OR = 0.91, 95% CI: 0.85-0.97; OR = 0.84, 95% CI: 0.78-0.90). Patients with a monthly salary of ≥57,801 NT$ have significantly fewer emergency department visits compared to those with a monthly salary of ≤17,280 NT$ (OR = 0.84, 95% CI: 0.77-0.93). Regarding the impact of treatment modalities for NSCLC, only surgical treatment (OR = 1.91, 95% CI: 1.43-2.57), only radiation therapy (OR = 1.61, 95% CI: 1.08-2.41), surgery + other treatments (OR = 1.93, 95% CI: 1.44-2.58), and chemotherapy + other treatments (OR = 1.48, 95% CI: 1.07-2.04) significantly increase emergency department visits compared to only targeted therapy. However, urbanization level of patients’ residential areas, severity of comorbidities (CCI), comorbid diseases, hospital ownership and level, patients’ experience with Chinese medicine, and year of diagnosis show no significant association with patients’ emergency department visits (p > 0.05), except for patients with comorbid chronic obstructive pulmonary disease, who significantly increase their use of emergency department visits (OR = 1.17, 95% CI: 1.02-1.35).

Using GEE multiple regression analysis to examine the association between the influence factors and the hospitalization days within 1 year of diagnosis for patients with NSCLC (Tables 5, 6), it was found that the average hospitalization days for NSCLC patients within 1 year was 32.01 ± 30.82. For patients using adjunctive TCHM therapy for 181–365 days, the average hospitalization days within 1 year decreased to 23.41 ± 21.43. Patients with NSCLC who used adjunctive TCHM therapy for 181–365 days had significantly shorter hospitalization days compared to those who did not receive adjunctive TCHM therapy (Ratio = 0.83, 95% CI: 0.75-0.91).

**TABLE 5:** Hospitalization days in NSCLC patients within 1 year of diagnosis.

Variable		Mean		SD	
Total		32.01		30.82	
Days of TCM					
	<30	32.36		31.32	
	30-180	32.70		29.51	
	181-365	23.41		21.43	
Age					
	20–54 y/o	33.73		32.75	
	55–64 y/o	31.84		30.68	
	65–74 y/o	31.72		29.96	
	≧75 y/o	28.59		27.10	
Gender					
	male	35.78		32.87	
	female	28.55		28.36	
Marital status					
	Unmarried	35.86		33.88	
	Married	31.45		30.38	
	Divorced/separated	37.12		35.11	
	Widowed	30.20		28.33	
Education level					
	Elementary school or below	32.19		29.77	
	Junior high school	35.19		33.15	
	High school (vocational)	32.54		32.25	
	College and above	28.02		28.61	
Monthly salary (NT$)					
	≦17,280	33.39		31.57	
	17,281–22,800	33.00		31.00	
	22,801–36,300	31.88		30.95	
	36,301–57,800	31.09		30.30	
	≧57,801	25.82		27.62	
Degree of urbanization					
	1	31.06		30.27	
	2	31.85		30.70	
	3	30.65		29.60	
	4	34.32		32.92	
	5	34.06		31.13	
CCI					
	0	32.07		30.84	
	1	31.30		32.38	
	≧2	28.40		25.90	
Comorbidities					
Hypertension					
	no		31.83		30.87
	yes		33.44		30.38
diabetes					
	no		31.81		30.75
	yes		36.90		32.08
hyperlipidemia					
	no		31.99		30.87
	yes		33.46		27.90
liver cirrhosis					
	no		31.96		30.78
	yes		42.64		36.21
CVD					
	no		31.91		30.82
	yes		40.07		29.69
COPD					
	no		31.81		30.69
	yes		44.30		35.39
asthma					
	no		31.91		30.72
	yes		39.95		37.03
renal failure					
	no		32.01		30.82
	yes		41.09		31.25
rheumatoid arthritis					
	No		32.01		30.81
	yes		34.13		33.46
chronic mental disorder					
	no		31.95		30.73
	yes		48.54		45.54
Hospital ownership					
	non-public		33.52		31.90
	public		29.68		28.91
Hospital level					
	medical centers		30.74		29.77
	regional hospitals		34.29		32.63
	district hospitals		37.87		32.14
Cancer stage					
	I		17.51		17.62
	II		26.29		23.10
	III		34.77		31.72
	IV		37.26		33.27
TCM experience					
	no		32.10		30.67
	yes		31.93		30.97
Treatment modalities					
	Surgery		27.59		29.05
	Chemotherapy		13.72		22.64
	Radiotherapy		24.73		27.42
	Targeted therapy		12.26		11.43
	Surgery + others		34.04		31.22
	Chemotherapy + others		28.24		36.76
	Other combinations		17.15		18.03

**TABLE 6:** GEE multiple regression analysis of hospitalization days in NSCLC patients within 1 year of diagnosis.

Variable		Ratio	95%CI		p value
Days of TCM					
	<30				
	30-180	1.03	0.97	1.09	0.333
	181-365	0.83	0.75	0.91	<0.001
Age					
	20-54 y/o				
	55–64 y/o	0.96	0.91	1.01	0.115
	65–74 y/o	0.93	0.87	0.99	0.019
	≧75 y/o	0.81	0.75	0.88	<0.001
Gender					
	male				
	female	0.76	0.72	0.79	<0.001
Marital status					
	Unmarried	1.18	1.08	1.30	<0.001
	Married				
	Divorced/separated	1.09	0.99	1.19	0.073
	Widowed	1.06	0.99	1.14	0.109
Education level					
	Elementary school or below				
	Junior high school	1.01	0.95	1.07	0.730
	High school (vocational)	0.95	0.90	1.01	0.083
	College and above	0.81	0.76	0.87	<0.001
Monthly salary (NT$)					
	≦17,280				
	17,281–22,800	0.92	0.87	0.98	0.009
	22,801–36,300	0.97	0.91	1.04	0.345
	36,301–57,800	0.92	0.86	0.99	0.020
	≧57,801	0.84	0.77	0.91	<0.001
Degree of urbanization					
	1				
	2	1.00	0.95	1.05	0.885
	3	1.00	0.94	1.06	0.995
	4	1.10	1.04	1.18	0.003
	5	1.08	1.00	1.17	0.041
CCI					
	0				
	1	1.14	0.98	1.33	0.084
	≧2	1.05	0.88	1.24	0.604
Comorbidities					
	hypertension	0.97	0.90	1.04	0.344
	diabetes	1.22	1.08	1.37	0.001
	hyperlipidemia	1.00	0.84	1.17	0.956
	liver cirrhosis	1.33	1.08	1.64	0.008
	renal failure	1.26	1.07	1.49	0.006
	CVD	1.35	1.16	1.57	<0.001
	COPD	1.12	0.90	1.39	0.295
	Asthma	1.03	0.78	1.37	0.825
	rheumatoid arthritis	1.18	0.85	1.64	0.323
	chronic mental disorder	1.61	1.20	2.16	0.002
Hospital ownership					
	non-public				
	public	1.05	1.00	1.09	0.054
Hospital level					
	medical centers				
	regional hospitals	1.07	1.02	1.12	0.003
	district hospitals	1.05	0.90	1.22	0.535
Cancer stage					
	I				
	II	1.52	1.39	1.67	<0.001
	III	1.80	1.70	1.92	<0.001
	IV	2.04	1.93	2.15	<0.001
TCM experience					
	no				
	Yes	1.05	1.01	1.09	0.021
Treatment modalities					
	Surgery	55.31	38.23	80.04	<0.001
	Chemotherapy	1.39	0.79	2.48	0.257
	Radiotherapy	5.63	2.98	10.63	<0.001
	Targeted therapy				
	Surgery + others	60.59	41.93	87.55	<0.001
	Chemotherapy + others	3.15	1.97	5.04	<0.001
	Other combinations	2.40	1.39	4.14	0.002
Years of diagnosis					
	2007				
	2008	1.00	0.92	1.08	0.955
	2009	0.99	0.92	1.07	0.765
	2010	0.95	0.88	1.03	0.227
	2011	0.90	0.83	0.97	0.006
	2012	0.80	0.74	0.86	<0.001
	2013	0.78	0.72	0.84	<0.001

Regarding other relevant factors, the longer the age of the patient, the shorter the hospitalization days. Compared to patients aged 20–54 years, patients aged 65–74 years have shorter hospitalization days (Ratio = 0.93, 95% CI: 0.87-0.99), and patients aged 75 years and above have even shorter hospitalization days (Ratio = 0.81, 95% CI: 0.75-0.88). Female patients have shorter hospitalization days within 1 year compared to male patients (Ratio = 0.76, 95% CI: 0.72-0.79), while unmarried patients have longer hospitalization days compared to married patients (Ratio = 1.18, 95% CI: 1.08-1.30). Patients with higher education levels have shorter hospitalization days; those with a college degree or above have significantly different hospitalization days compared to those with elementary school education or below (Ratio = 0.81, 95% CI: 0.76-0.87). Similarly, patients with higher monthly salaries also have shorter hospitalization days; for example, patients with a monthly salary of ≥57,801 NT$ have shorter hospitalization days compared to those with a monthly salary of ≤17,280 NT$ (Ratio = 0.84, 95% CI: 0.77-0.91). Patients living in less urbanized areas have longer hospitalization days; for example, patients in level 5 urbanization areas have longer hospitalization days compared to those in level 1 urbanization areas (Ratio = 1.08, 95% CI: 1.00-1.17). The CCI does not affect the hospitalization days, but patients with comorbidities such as diabetes (Ratio = 1.22, 95% CI: 1.08-1.37), cirrhosis (Ratio = 1.33, 95% CI: 1.08-1.64), cerebrovascular disease (Ratio = 1.26, 95% CI: 1.07-1.49), chronic obstructive pulmonary disease (Ratio = 1.35, 95% CI: 1.16-1.57), and chronic mental illness (Ratio = 1.61, 95% CI: 1.20-2.16) have significantly longer hospitalization days. The ownership of hospitals (public, non-public) does not affect the hospitalization days for NSCLC patients, but the hospital level does. Patients treated at regional hospitals have longer hospitalization days compared to those treated at medical centers (Ratio = 1.07, 95% CI: 1.02-1.12). The higher the cancer stage of NSCLC patients, the longer the hospitalization days. Compared to stage I patients, stage II patients have a ratio of 1.52 (95% CI: 1.39-1.67), stage III patients have a ratio of 1.80 (95% CI: 1.70-1.92), and stage IV patients have a ratio of 2.04 (95% CI: 1.93-2.15) of hospitalization days. Patients with experience using Chinese medicine have longer hospitalization days compared to those without experience (ratio = 1.05, 95% CI: 1.01-1.09). In terms of treatment combinations, patients receiving pure targeted therapy have the shortest hospitalization days. Patients undergoing surgical treatment have longer hospitalization days, with pure surgical patients having a ratio of 55.31 (95% CI: 38.23-80.04) and patients receiving surgery + other treatments having a ratio of 60.59 (95% CI: 41.93-87.55). Patients receiving pure radiation therapy also have longer hospitalization days (ratio = 5.63, 95% CI: 2.98-10.63), as do patients receiving chemotherapy + other treatments (ratio = 3.15, 95% CI: 1.97-5.04) and other treatment combinations (ratio = 2.40, 95% CI: 1.39-4.14). As for the year of cancer diagnosis, the later the diagnosis, the shorter the hospitalization days; for example, compared to patients diagnosed in 2007, patients diagnosed in 2013 have a ratio of 0.78 (95% CI: 0.72-0.84).

## Discussion

The results of this study indicate that many factors are associated with the use of TCHM as adjunctive therapy, including cancer stage, age, gender, marital status, education level, monthly income, urbanization level of residence area, CCI, comorbid diseases, hospital ownership, experience with TCM, receipt of chemotherapy, and year of diagnosis. Several factors are consistent with previous research findings (Lai et al., 2017; Lin et al., 2008; Lin et al., 2016; Lu et al., 2019; Weng et al., 2016), such as patients undergoing chemotherapy, female patients compared to male patients, younger age groups, higher severity of comorbidities, presence of multiple comorbidities, higher income levels, and residence in urban areas having a higher probability of using TCM treatment. However, some results differ from previous studies (Sun et al., 2018). For example, this study found that patients in the early stages of cancer are not more likely to seek TCM treatment, possibly because early-stage NSCLC can be treated with surgery and cured, which may explain why patients in Taiwan with early-stage NSCLC may not necessarily use TCM. The level of hospital and the receipt of TCM treatment are not associated, but NSCLC patients in non-public hospitals are more likely to receive TCM treatment than those in public hospitals. It is speculated that patients in hospitals with TCM departments can conveniently receive TCM treatment nearby, and Western medicine departments are more likely to consult TCM. Hospitals with TCM departments are more common in non-public hospitals than in public hospitals (Ministry of Health, 2023). This study’s data is linked to the household registration database from the Ministry of the Interior, and compared to previous studies, it includes marital status and education level as variables. Married individuals and those with higher education levels have a higher probability of receiving TCM treatment, highlighting this study’s significance, particularly as it is a nationwide cohort study.

This study examines the commonly used TCHM single herbs and formulas among NSCLC patients, with results differing slightly from previous research. A study from Taiwan in 2017 reported that the most commonly used formula was Qing Zao Jiu Fei Tang (Liao et al., 2017), which differs from the findings of this study, where San Jhong Kuei Jian Tang was most common. The reason for this difference may be attributed to the disparity in study periods. The previous study analyzed data from the NHIRD from 2000 to 2009, whereas this study covers the years 2007–2013, resulting in significant overlapping differences in time. San Jhong Kuei Jian Tang is known for its efficacy in clearing heat, detoxification, and resolving masses and swelling; it is often used to treat complications of NSCLC, lymphadenitis, acute folliculitis, thyroiditis, and thyroid nodules (Chinese Medicine formulae, 2024a). On the other hand, Qing Zao Jiu Fei Tang is known for clearing dryness and nourishing the lungs, suitable for conditions such as pneumonia, pulmonary tuberculosis, bronchial asthma, acute and chronic bronchitis, emphysema, lung cancer, urticaria, and laryngitis (Chinese Medicine formulae, 2024b). Based on the aforementioned, it can be inferred that San Jhong Kuei Jian Tang is mainly used for complications associated with NSCLC, while Qing Zao Jiu Fei Tang is employed as an anticancer formula. Moreover, a Taiwanese study from 2020 reported results consistent with this study (Yeh et al., 2020), where Hedyotis diffusa was the most commonly used single herb. Hedyotis diffusa is known for its efficacy in clearing heat, detoxification, promoting urination, and having antibacterial and anti-inflammatory properties. It also exhibits anti-tumor effects, inhibits spermatogenesis, and has hepatoprotective and choleretic effects (Ministry of Agriculture, 2024).

Among the top ten commonly used single herbs and formulas in this study for NSCLC patients receiving adjunctive TCHM therapy, herbs such as Hedyotis diffusa, Platycodon root, Astragalus root, Tendrilleaf fritillary bulb, Heartleaf houttuynia, Skullcap, Scutellaria root, Bitter apricot seed, Rhubarb, and Mongolian dandelion are all associated with lung cancer treatment. Among the formulas, Siang Sha Liou Jyun Zih Tang, Bai He Gu Jin Tang, Qing Zao Jiu Fei Tang, Bu Zhong Yi Qi Tang, and Ganlu Yin are related to anticancer effects. Additionally, formulas like San Jhong Kuei Jian Tang, Xiao Chai Hu Tang, Jiawei Siaoyao San, Bansia Siexin Tang, and Wen Dan Tang exhibit anti-inflammatory, immune-enhancing, and digestive system-strengthening effects, which can alleviate complications of NSCLC and side effects of Western medication (Zhang et al., 2021; Su et al., 2020; Lo and Hung, 2021).

The adjunctive TCHM therapy for 181–365 days in patients with NSCLC can significantly reduce emergency department visits by 16% and hospitalization days by 17%. The adjunctive TCHM therapy can improve medical utilization in NSCLC patients, provided that the duration of TCHM use reaches 181–365 days. Previous literature has not studied the emergency department visits and hospitalization days in the NSCLC patients treated with adjunctive TCHM therapy. Therefore, our study has reached a new conclusion and cannot be compared with other similar studies. This crucial finding can provide evidence-based recommendations for clinicians, indicating that TCHM must be used for at least 6 months to significantly reduce emergency department visits and hospitalization days. In other words, the adjunctive TCHM therapy of NSCLC should focus on the “course of treatment” rather than just the dosage effect. Even though the life expectancy of patients with advanced stage NSCLC may be shorter, this study showed that the benefits of adjunctive TCHM therapy were more significant in patients with advanced stage NSCLC, and patients with advanced stage NSCLC should be encouraged to receive the adjunctive TCHM therapy.

In addition to other relevant factors affecting emergency department visits, patients with more advanced cancer stages, those aged 75 and above, males, individuals with lower education levels, those with comorbid chronic obstructive pulmonary disease (COPD), and patients undergoing surgery or chemotherapy tend to have higher rates of emergency department visits. The findings of this study regarding gender, age, and income align with a study from mainland China in 2021 (Zhu et al., 2021), but differ from a study conducted by American scholars in 2008 regarding gender (Shugarman et al., 2008). However, the medical utilization patterns of lung cancer patients with comorbidities in mainland China, as observed in a 2020 study, differ from those in this study. In Taiwan, only comorbid COPD increases emergency department visits among NSCLC patients, while the mainland Chinese study found that several comorbid conditions were associated with medical utilization (Ding et al., 2020).

In terms of other relevant factors affecting hospitalization days, patients with more advanced cancer stages, males, those with lower education levels, individuals with comorbid chronic obstructive pulmonary disease (COPD), and patients undergoing surgery tend to have longer hospitalization days, in addition to higher rates of emergency department visits. However, some factors show different patterns compared to emergency department visits. For instance, patients aged 75 and above tend to have shorter hospitalization days compared to other age groups. Previous literature often focused on the frequency of hospitalizations among NSCLC patients rather than the duration, making direct comparisons difficult (Zhu et al., 2021). This could be attributed to the higher mortality rate among patients aged 75 and above, leading to shorter average hospitalization days. Patients with multiple comorbidities, including diabetes, liver cirrhosis, cerebrovascular diseases, and chronic mental disorders, significantly increase hospitalization days. While these four comorbidities do not increase the emergency department visit, they do prolong hospitalization days, providing crucial indicators for clinical caregivers. Regarding treatment combinations, only pure targeted therapy and pure chemotherapy do not significantly increase hospitalization days, while other treatments may require hospitalization or pose an increased risk of hospitalization.

### Strengths

This study has several strengths. Firstly, it utilizes nationwide statistical data, encompassing 7 years of lung cancer patients (2007-2013). Secondly, the study employs Propensity Score Matching (PSM) with a 1:5 ratio to control for variables such as cancer stage, gender, age, monthly income, education level, marital status, disease severity, medical history (diabetes, liver cirrhosis, renal failure, cerebrovascular diseases, chronic obstructive pulmonary disease), and whether surgery was performed within 90 days. This meticulous matching ensures precise comparison between patients receiving and not receiving adjunctive TCHM therapy for lung cancer, effectively addressing potential confounders.

### Limitations

This study’s sample was obtained from the NHIRD, and when analyzing based on diagnosis codes and medical order codes, the real-world compliance of patients with TCHM and Western medicine was overlooked. Retrospective studies may encounter unmeasured confounders (such as BMI, smoking status, or alcohol drinking) that might potentially impact the analysis results. The administration data used in retrospective studies may have incomplete data, data quality issues, temporal bias, or a lack of some relevant variables, making it impossible to completely reflect the current clinical situation. Additionally, data on certain TCHM treatments that are paid out-of-pocket (such as a few herbal decoctions) are not available in the NHIRD. If these out-of-pocket TCM treatments contribute to reduced medical utilization, the study also included their effects in the analysis for comparison. The out-of-pocket herbal decoctions will not affect the results because they were part of the TCM group. Each country’s medical system and economic environment were different; the results of this study might not be applicable outside of Taiwan. Finally, this study explored association between main outcomes and relevant variables and did not focus on examining causal inference.

## Conclusion

Patients with NSCLC who receive adjunctive TCHM therapy for 181–365 days could significantly reduce emergency department visits and shorten hospitalization days. Clinical treatment teams may recommend and encourage patients with late-stage cancer, older age, male gender, low education level, and low income to undergo adjunctive TCHM therapy for at least 6 months. We strongly suggest the need for more research to confirm the findings.

## Data Availability

This study used databases including the National Health Insurance Research Database and the Cause of Death File. Data are available from the National Health Insurance Research Database published and managed by the Ministry of Health and Welfare, Taiwan. Due to legal restrictions imposed by the Taiwan government related to the Personal Information Protection Act, these databases cannot be made publicly available. All researchers can apply to access the databases for the purpose of conducting their studies. Requests for using the data can be sent as a formal proposal to the Science Center of the Ministry of Health and Welfare (https://www.mohw.gov.tw/mp-2.html). Any raw data are not allowed to be brought out from the Science Center. Only the analytic outputs in the format of a table or figure can be printed out. These restrictions prohibit the authors from making the minimal datasets publicly available. All remaining data are available in the article. Further inquiries can be directed to the corresponding author(s).

## Abbreviations

NSCLC, Non-Small Cell Lung Cancer; NHIRD, National Health Insurance Research Database; ICD, International Classification of Diseases; CCI, Charlson Comorbidity Index; CI, confidence interval; TCM, Traditional Chinese Medicine; TCHM, Traditional Chinese Herbal Medication; OR, odds ratio.
